# Comparative assessment of hemodynamic changes and outcomes in ventilator weaning

**DOI:** 10.2478/jccm-2025-0022

**Published:** 2025-07-31

**Authors:** Dina Zeid Roushdy, Hossam Ahmed Saad, Randa Aly Soliman, Mohammed Aly Shehata, Mohammed Amin Fakhir

**Affiliations:** Cairo University Kasr Alainy Faculty of Medicine, Cairo, Egipt

**Keywords:** mechanical ventilation, hemodynamic changes, volume-controlled ventilation, variable pressure support, pressure support

## Abstract

**Introduction:**

Mechanical ventilation is fundamental for the management of critically ill patients. The mode of mechanical ventilation may impact the patient in different ways. This study aimed to assess the hemodynamic changes occurring when transitioning between Volume-Controlled Ventilation (VCV) to Variable Pressure Support (VPS) and VCV to Pressure Support (PS) by echocardiography. Also, a comparison between the spontaneous breathing modes in terms of outcomes, specifically, weaning of mechanical ventilation, days on a ventilator, hospital days, and survival, was conducted.

**Methods:**

This prospective observational study was conducted on 40 mechanically ventilated patients who showed readiness for weaning from Mechanical ventilation. When transitioning between VCV and VPS in arm A and from VCV to PS in arm B, an echocardiographic assessment (transesophageal echocardiography and transthoracic echocardiography) was performed. Both modes were further compared in terms of weaning and the success of liberation from mechanical ventilation.

**Results:**

By comparing both arms, there was a significant difference in velocity time integral (VTI) and stroke volume (SV) for TEE and TTE with p-values of 0.044, 0.022, and 0.05, 0.059, respectively. Also, the cardiac output (CO) showed a statistically significant difference between both arms with a p-value of 0.04. On the other side, there was no statistically significant difference between both arms in terms of ventilator days (p-value of 0.88), length of stay (p-value of 0.651), weaning trial success (p-value of 0.525), and survival rate (p-value of 0.525).

**Conclusion:**

The study showed that VPS is a promising modality that can be used in place of PS as a weaning mode. It provides better patient comfort and a more physiological way of breath delivery. The study also concluded that TTE and TEE will show similar results in most patients and that both can be used interchangeably.

## Introduction

Mechanical ventilation is a vital parameter in intensive care medicine. It has served as a bridging therapy for compromised patients to alleviate unneeded pulmonary exhaustion and allowed for smooth neurological, cardiac, and pulmonary recovery with minimal complications [1]. However, the disadvantages, including the hemodynamic effects of mechanical ventilation, have been studied thoroughly, and the emphasis on preload, afterload, and contractility affection by positive pressure ventilation (PPV) can't be overstated [2,3].

Mechanical ventilation can be delivered through many interfaces, whether invasive or non-invasive [4,5]. Controlled modes of mechanical ventilation occur when the ventilator administers a predetermined volume or pressure, irrespective of the patient's inspiratory efforts. Assist-control ventilation suits individuals who are not deeply sedated and can initiate breathing but not sustain spontaneous respiration [6]. However, controlled modes are associated with fixed volumes and/or pressures, which can increase intrathoracic pressure (ITP) and impede venous return and, thus, cardiac output. Also, patient-ventilator desynchrony is a significant side effect [7].

Variable pressure support ventilation (VPS) is a form of assisted ventilation that adjusts the level of pressure support on a breath-by-breath basis to re-establish the physiological variability of respiratory activity [8]. VPS emulates the delicate diversity of regular respiration by producing random fluctuations in inspiratory pressure. The primary potential outcomes of this mild modification are enhanced pulmonary function and a diminished risk of ventilator-associated lung injury [9]. Patients also feel more comfortable with minimal adjustments to the ventilator settings: more homogenous ventilation, surfactant production, and pulmonary perfusion without adverse hemodynamic effects of elevated intrathoracic pressures [7].

Transesophageal echocardiography (TEE) has emerged as a valuable diagnostic and monitoring instrument in critical care environments, mainly where transthoracic echocardiography is challenging to execute [10]. It offers essential information concerning mechanical ventilation in specific populations, such as obese individuals and those with surgical dressings and chest tubes. TEE can also be performed in a prone position [11,12]. It also enables visualization of the superior vena cava, which can aid in predicting fluid response in mechanically ventilated patients [7].

Transthoracic echocardiography (TTE) is among the most frequently conducted cardiac assessments. It offers extensive information regarding heart structure and function, aiding diagnosis and therapeutic guidance, and is no longer exclusive to specialist cardiology departments [13]. While TTE is mainly utilized to investigate heart function, its applications in hemodynamic evaluations are gaining popularity. Estimates of cardiac output, proper atrial pressure, and assessments of patients' fluid status are prevalent applications of the TTE [14].

This study will use TEE and TTE to compare the hemodynamic profiles of VPS and Pressure Support (PS) modes to those of the Controlled mode of mechanical ventilation, volume-controlled ventilation (VCV), and compare weaning outcomes and survival in both.

## Methods

### Patients

This prospective cohort observational study was performed on 40 intubated and mechanically ventilated patients hospitalized at the Critical Care Department-Cairo University Hospital from February 2023 to June 2024. These patients were ready for liberation from mechanical ventilation and showed readiness for weaning. The Local Research Ethics Committee (REC) for experimental and clinical studies at the Critical Care Department of the Faculty of Medicine, Cairo University, approved this research protocol (MD-204-2022). Informed consent was acquired from the first-degree relatives of the patients, who were apprised of the method. Our research adhered to the principles of the Declaration of Helsinki.

To fit the inclusion criteria for this study, firstly, patients were monitored on assisted-controlled mechanical ventilation for a maximum of 48 hours prior to being deemed prepared for a first spontaneous breathing experiment [15]. Then, patients were included in the weaning trial if they met the following: adult patients > 18 years, improvement of the underlying cause of invasive mechanical ventilation, body temperature < 39°C, hemoglobin level > 7g/dl, PaO2 > 60 mmHg, FIO2 ≤ 40%, PEEP ≤ 8 cm H2O, respiratory rate was less than 35 breaths/minute, systolic arterial pressure > 90 mmHg (without need for/or high dose vasoactive drugs) and < 160 mmHg, no sedation, and stable neurological status.

On the other side, the exclusion criteria comprised patients dependent on high FiO2 >0.5, patients who needed high PEEP (>10cm H2O), a PaO2/FiO2 ratio less than 150, minute ventilation requirement more significant than 15 L per minute, rapid shallow breathing index over 105 (shallow rapid breaths with higher respiratory rate and lower tidal volumes), patients with impaired consciousness who cannot protect their airways, and patients on high doses of vasopressor and/or inotropic support. Also, patients diagnosed with severe neuromuscular disorders, arrhythmic patients, patients with impaired cardiac Systolic function (EF<45%), and patients with contraindications for TEE placement (for example, gastroesophageal obstruction, recent gastroesophageal surgeries, or others) were excluded [16].

Patients who met the inclusion/exclusion criteria were divided randomly into two arms: arm-A (VPS group) and arm-B (PS group). VCV was the initial mode in both arms, then switched to VPS in arm-A or PS in arm-B. The randomization was done by assembling 40 indistinguishable envelopes, half including a label designating the “VPS” group and the other half containing a label designating the “PS” group and providing detailed instructions. The investigator prepared and sealed all envelopes before commencing enrollment. Upon enrollment, every participant had the opportunity to select one envelope, which would decide their assigned group. Both groups were compared in terms of days on mechanical ventilation, success of liberation from mechanical ventilation, days in ICU, and mortality.

### Procedures

A complete medical history was taken from the eligible patients after their first-degree relatives signed an informed consent form. Afterward, patients were subjected to clinical examination (body weight, height, BMI, head/neck assessment, upper/lower limb and abdominal examination, and chest/cardiac evaluation). Mechanical ventilation was examined using an Evita® V600 mechanical ventilator (Germany). An echocardiographic evaluation was done on assisted controlled ventilation at the end of VPS and standard PS using a Philips iU22 Ultrasound System (United States) [17]. It was performed 15 minutes later after the mode change from VCV to VPS or VCV to PS modes to assess the immediate hemodynamic effects [18]. This was standardized throughout the study to allow enough time between modes and to accommodate changes in intrathoracic pressures that were expected to occur during the transitioning between the modes. TTE was conducted first, followed by TEE, to allow for a comprehensive assessment of cardiac function [19,20] and to prioritize non-invasive assessment with TTE before proceeding to TEE [21]. TTE examinations were recorded, including M-mode, two-dimensional (2D), Color flow mapping, and Doppler measurements. After the TTE examination, a TEE examination will be performed on the same ultrasound system using a TEE probe (PHILIPS X8-2t Ultrasound Transducer) [22].

To reduce any potential operator bias during the echocardiographic assessments, the operators were blinded to the patients' groups (VPS or PS), and the assessors of the echocardiography results were blinded during the data analysis. Also, the operators, the assessors, the devices, and the techniques were fixed during the assessment. Additionally, all the operators are well-trained and certified in using echocardiographic techniques with full adherence to echocardiographic protocols.

The hemodynamic assessment was done, and the patients were assessed for weaning eligibility later. Patients who succeeded in passing SBT were then extubated with continuous monitoring and observation for signs of failure of weaning for 48 hours. Failure of the weaning process was defined as a failed SBT or the need for reintubation within 48 hours following extubation [23]. Any indicators of Weaning failure were recorded as follows [24]: arterial oxygenation saturation (SaO2) <85% – 90%, pH < 7.32, increase in PaCO_2_ >10 mmHg during SBT, respiratory rate >30 – 35 breaths/minute, and respiratory rate change over 50%. At the same time, CVS indicators included heart rate >120 – 140 beats/minute, systolic blood pressure >180 mmHg or <90 mmHg, any change in mean arterial pressure (MAP) more significant than 20%, and the need for vasopressors required. Other indicators like nasal flaring, accessory respiratory muscles, paradoxical breathing movement, altered mental status, and agitation were also observed [24].

### Statistical Analysis

The data was encoded and input utilizing the Statistical Package for the Social Sciences (SPSS) version 25 (IBM Corp., Armonk, NY, USA). The subsequent metrics were used to assess quantitative data: mean, standard deviation, median, minimum, and maximum; frequency (count) and relative frequency (%) were employed to evaluate categorical data. The non-parametric Kruskal-Wallis and Mann-Whitney tests were utilized to compare quantitative variables. Non-parametric Friedman and Wilcoxon signed-rank tests were used to compare serial measurements within each patient (Chan, 2003a). The Chi-square test was employed to analyze categorical data. For multiple comparisons, the Spearman correlation coefficient (Chan, 2003c) and a post-hoc analysis were used. P-values less than 0.05 were considered statistically significant. Multivariable Regression Analysis (linear regression) was utilized for multiple confounders by including them as covariates in the model [25].

## Results

The mean age of the enrolled patients was 55.55±28.7 years, where age showed a statistical difference between both arms of patients with a mean value of 62.2 in arm-A and 48.9 in arm-B with a p-value of 0.028. As shown in Table 1, males were predominant, representing 65% of the included individuals, while 35% were females. There was no statistical significance regarding gender between the two arms (p-value=1). The prevalence of comorbidities is represented in Table 1; only Diabetes Mellitus showed a statistical significance between arm-A and arm-B with a p-value of 0.047.

**Table 1.** Patient characteristics and data during the study

**Data**		**Arm-A (VPS group)**	**Arm-B (PS group)**	**p-value**
Gender	Male	13 (65%)	13 (65%)	1
	Female	7 (35%)	7 (35%)	

Co-morbidities				
Hypertension		13 (65%)	8 (40%)	0.113
Diabetes Mellitus		10 (50%)	4 (20%)	0.047
Chronic Kidney Diseases		2 (10%)	5 (25%)	0.407
Ischemic Heart disease		4 (20%)	3 (15%)	1
Chronic obstructive pulmonary disease		3 (15%)	2 (10%)	1

Clinical Data (mean± S.D)				
HR		88.35±19.98	882.65±13.55	0.298
MAP		73.40±7.76	70.20±7.17	0.183

Echocardiographic Data (mean± S.D)				
LVOT diameter (cm)		2.05±0.33	1.93±0.30	0.20
TEE VTI (VCV)		20.50±2.74	21.70±3.26	0.223
TTE VTI (VCV)		20.50±2.91	22.10±3.11	0.101
TEE SV (VCV)		67.95±18.63	63.95±16.87	0.481
TTE SV (VCV)		68.00±18.17	63.90±16.74	0.463
CO (VCV)		5.74±1.34	5.39±1.73	0.473

CO: cardiac output; HR: heart rate; LVOT: left ventricular outflow tract; MAP: mean arterial pressure; PS: pressure support; SV: Stroke volume; VCV: Volume-Controlled Ventilation; VPS: variable pressure support; VTI: velocity time integral

Throughout the hemodynamic assessment (using TTE and TEE), both vital signs, heart rate, and mean arterial pressure changes didn't show statistical differences between the two arms. For the echocardiographic data, the left ventricular outflow tract (LVOT) diameter didn't show any statistical significance between the two arms, with a mean value of 2.05 cm in arm-A and 1.93 cm in arm-B with a p-value of 0.2. Comparing the two arms in terms of hemodynamic parameters under VCV (stroke volume SV, velocity time integral VTI, and cardiac output CO) measured by TTE and TEE, there was no statistically significant difference, as shown in Table 1.

**Fig. 1.**
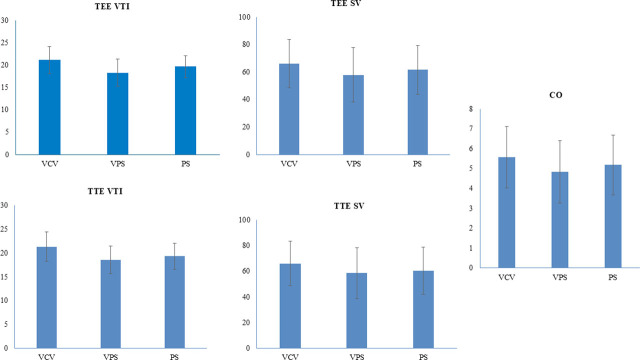
**Intercomparison of hemodynamic parameters between the VTI, SV, and CO.** CO: cardiac output; SV: stroke volume; TEE: transesophageal echocardiography; TTE: transthoracic echocardiography; VCV: volume-controlled ventilation; VPS: variable pressure support; VTI: velocity time integral

The inter-statistical comparison between the three modes revealed that in arm-A, there was statistical significance between VCV and VPS modes with drops in VTI, SV, and CO with p-values of TEE VTI, and TEE SV were 0.02 and 0.03. TTE VTI and TTE SV were 0.04,0.05, respectively. CO drop was also significant, with a p-value of 0.01 (Table 2). Statistical analysis depicted a decline of 13% in mean TEE and mean TTE VTI when the change from VCV to VPS was done, which translated to subsequent drops in mean SV and mean CO by 12.1 % and 13%, respectively (Figure 1).

**Table 2.** Inter-statistical comparison between the three modes in both arms

**Parameter**	**VCV (mean ± SD)**	**VPS (mean ± SD)**	**p-value**
TEE VTI	21.11±3.03	18.30±3.07	0.02
TTE VTI	21.30±3.08	18.53±2.95	0.04
TEE SV	65.95±17.66	57.93±19.73	0.03
TTE SV	65.95±17.37	58.48±19.88	0.05
CO	5.57±1.54	4.83±1.58	0.01

Parameter	VCV (mean ± S.D)	PS (mean ± S.D)	p-value
TEE VTI	21.11±3.03	19.65±2.48	0.04
TTE VTI	21.30±3.08	19.33±2.74	0.05
TEE SV	65.95±17.66	61.58±17.87	0.03
TTE SV	65.95±17.37	60.45±18.54	0.02
CO	5.57±1.54	5.18±1.50	0.04

Parameter	VPS (mean ± S.D)	PS (mean ± S.D)	p-value
TEE VTI	18.30±3.07	19.65±2.48	0.004
TTE VTI	18.53±2.95	19.33±2.74	0.05
TEE SV	57.93±19.73	61.58±17.87	0.002
TTE SV	58.48±19.88	60.45±18.54	0.059
CO	4.83±1.58	5.18±1.50	0.04

CO: cardiac output; PS: pressure support; SV: stroke volume; TEE: trans-esophageal echocardiography; TTE: transthoracic echocardiography; VCV: volume-controlled ventilation; VPS: variable pressure support; VTI: velocity time integral

In group B, there was statistical significance between VCV and PS modes with drops in VTI, SV, and CO with p-values of TEE VTI and TEE SV were 0.04 and 0.03. TTE VTI and TTE SV were 0.05,0.02, respectively. CO drop was also significant, with a p-value of 0.04 (Table 2). Statistical analysis depicted a decrease of 6% in mean TEE and mean TTE VTI when the change from VCV to PS was done, which translated to subsequent drops in mean SV and mean CO by 6 % and 3.8%, respectively (Figure 1).

As shown in Table 3, comparing the use of TTE and TEE for the detection of VTI in both groups, there wasn't any statistical significance between using TTE or TEE in detecting VTI with a p-value in VCV, PS, and VPS of 0.313, 0.202, and 0.108, respectively.

**Table 3.** Analysis of TTE and TEE

**Ventilation mode**	**Parameter**	**TEE (mean ± S.D)**	**TTE (mean ± S.D)**	**p-value**
VCV	VTI	21.11±3.03	21.30±3.08	0.313
	SV	65.95±17.66	65.95±17.37	1

PS	VTI	19.65±2.48	19.33±2.74	0.202
	SV	61.58±17.87	60.45±18.54	0.356

VPS	VTI	18.30±3.07	18.53±2.95	0.108
	SV	57.93±19.73	58.48±19.88	0.079

PS: pressure support; SV: stroke volume; TEE: trans-esophageal echocardiography; TTE: transthoracic echocardiography; VCV: volume-controlled ventilation; VPS: variable pressure support; VTI: velocity time integral

For the hemodynamic assessment, the patients were randomly assigned to arm A (left to continue weaning in VPS mode) and arm B (left to continue weaning in PS mode). The analysis revealed no statistical significance between both groups regarding ventilator days (p-value of 0.88). Weaning trial success was also statistically insignificant, showing a p-value of 0.525. Likewise, the length of stay and survival showed no statistically significant difference between the two arms, as shown in Table 4.

**Table 4.** The Outcomes Statistics of the hemodynamic assessment between the two arms

**Outcomes**		**Arm-A (VPS group)**	**Arm-B (PS group)**	**p-value**
Ventilator days (mean ± S.D)		9.05± 4.56	9.30± 5.97	0.882
Length of stay (mean ± S.D)		16.20±6.92	17.25±7.64	0.651

Weaning trial	Yes (n, %)	12 (60%)	10 (50%)	0.525
	No (n, %)	8 (40%)	10 (50%)	

Survival	Yes (n, %)	10 (50%)	8 (40%)	0.525
	No (n, %)	10 (50%)	12 (60%)	

VPS: variable pressure support; VTI: velocity time integral

## Discussion

Mechanical ventilation provided for each patient can be done in various modes, whether controlled/assisted or spontaneous, and these three general outlines are further subdivided into a diverse panel of modes, each with an advantage to suit a particular patient [26,27]. An assessment of the disadvantages of the different modes that support the compromised state of the typical ICU patient is needed. This study was conducted on 40 mechanically ventilated patients who met the inclusion criteria of this study and then further subdivided into two arms: arm A comprised of patients receiving VPS, and arm B comprised of patients receiving PS. This was done by TTE and TEE of the patients, determining LVOT and VTI to calculate CO.

In our study, a notable difference in baseline characteristics between the treatment arms, particularly in age and the prevalence of diabetes, was observed. The average age of participants in the VPS group was significantly higher than in the control group (mean age 62.2 vs. 48.9 years, p < 0.05), and a greater proportion of participants in the VPS group had a diagnosis of diabetes (50% vs. 20%, p < 0.05). These differences are critical to consider as they may influence the outcomes of interest, including hemodynamic parameters, ventilator days, and overall clinical recovery. There was no statistical significance regarding gender between the two arms (p-value=1). The prevalence of comorbidities is represented in Table 1.

Age is a well-established factor that can affect physiological responses and recovery trajectories in critically ill patients. Older adults often exhibit altered hemodynamic responses and may have a higher prevalence of comorbid conditions that can complicate their clinical course [28,29]. The significant age difference between the groups may contribute to the observed variability in outcomes, particularly in the VPS group, which exhibited greater declines in VTI, SV, and CO. These hemodynamic changes, while statistically significant, didn't affect the clinical relevance in older patients who have different baseline physiological reserves compared to younger patients [30]. The presence of diabetes is another critical factor that may affect recovery and outcomes. Diabetes can lead to microvascular complications and impaired hemodynamic regulation, potentially exacerbating the impact of hemodynamic changes on clinical outcomes [31]. The interaction between diabetes and hemodynamic stability may warrant further investigation [32], as it could explain some of the differences in clinical outcomes observed between the groups. To address these baseline differences, we employed multivariable regression analysis to adjust for age and diabetes status in our primary outcome assessments.

In our study, we found out that there was a drop in VTI (which affected SV and CO) when changing from VCV to VPS mode in the majority of arm A patients, with a significant statistical p-value of 0.02 and was measured to be a 13% drop in LVOT VTI. This agrees with a study by Carlos et al. [33], which showed that PPV can harm the circulatory system, whether on the extremely sensitive right ventricle, a borderline function left ventricle, or in a preload-dependent state [33]. Likewise, Lai et al. [34] deduced that PEEP levels had a deleterious impact when increased in the circulatory system. Also, Vignon et al. [35] revealed that TEE is a valuable, well-tolerated imaging technique used in mechanically ventilated patients to assess left ventricular systolic function in the presence of PEEP [35].

This was controversial to Naik et al. [36], who revealed that VPS (or as alternately named noisy PS) had no adverse effects on hemodynamics [36]. Furthermore, Spieth et al. [37] claimed that VPS mode had no significant adverse hemodynamic effects compared to the controlled mode [37]. Discrepancies between this study and Spieth et al. could be attributed to one of three reasons. Firstly, in our study, maximum variability was adjusted with a range of pressures from 10 cmH2O to 20 cmH2O, which provided a wide range of pressures, some of which can generate supra-normal volumes [37]. Secondly, the baseline volume status differs from patient to patient and could be affected by multiple constantly changing factors. Finally, patients experienced dyspnea when switched from controlled modes to spontaneous ones, which could negatively affect certain patients, especially those with ischemic heart disease, and those constituted 17.5% of the total study population.

This research detected a less significant drop in most arm B patients when transitioning from VCV to PS mode, with a 6% drop in LVOT VTI and a statistically significant p-value of 0.04. These findings were similar to those of Mauri et al. [38], who reported that there is a potential risk of double and reverse triggering during the spontaneous modes of mechanical ventilation, which could lead to the delivery of non-protective volumes to the patient with a massive swing in intrathoracic pressures with a direct effect on the right ventricle [38]. Similarly, Frazier et al. [39] found a drop in MAP and CO when patients transitioned from controlled to spontaneous mechanical ventilation [39].

On the contrary, EL Gazzar et al. [40] showed a statistically significant reduction in diaphragmatic performance in patients who received controlled mechanical ventilation alone. Conversely, diaphragmatic performance improved when pressure support ventilation (PSV) was combined with cytomegaly virus, with no significant alterations in diaphragmatic mobility parameters. No significant link was observed between echocardiographic measures (left ventricular ejection fraction, right ventricular size, tricuspid annular plane systolic excursion, and proper ventricular systolic pressure) and various mechanical ventilation [40].

No consensus has been reached regarding using TTE or TEE to assess hemodynamics during PPV. For instance, in 2019, Tongyoo et al. used TTE for the evaluation of hemodynamics [41], while back in 1993, Poelaert et al. [42] used TEE in the hemodynamic assessment of mechanically ventilated patients [42]. Further Oh. et al. studied critically ill patients using TEE to assess intravascular volume [43]. The authors found that the predominant indication, observed in 25 individuals (49%), was unexplained hemodynamic instability. TEE indicated cardiovascular issues in 30 patients (59%) that were not distinctly diagnosed by TTE. Meanwhile, TEE enabled the definitive exclusion of suspected anomalies due to its enhanced imaging capabilities. Cardiac surgery was necessitated by TEE results in 12 individuals (24%), all of which were corroborated during the operation [43].

In this study, the authors primarily chose to use TEE due to the presence of patients with poor TTE windows, as in COPD patients, which constituted 12.5% of the patients. Furthermore, obese patients and others with skeletal deformities also had obscure echocardiographic windows and bad images. TEE offered a more detailed and less interpatient variability of echo images. Nevertheless, TTE and TEE didn't show any statistically significant values with almost identical p-values in VCV, PS, and VPS of 0.313, 0.202, and 0.108, respectively. In alignment with this study, Si et al. [44] concluded that TEE alone or in combination with TTE could provide valuable information for diagnosis, which may bring significant therapeutic benefits, as 66% of their patients had the same findings in both TTE and TEE with no change in therapy [44].

After hemodynamic assessment, the patients in arm A(VPS) and arm B (PS) were left for weaning in their respective modes. Both modes didn't show a statistically significant p-value regarding days on mechanical ventilation and liberation success, with p-values of 0.88 and 0.525, respectively. Our results are concordant with those of Mauri et al. [45], who revealed that VPS led to even and homogenous air distribution and was equally effective as PS in weaning [45]. Moreover, Wysocki et al. [46] have deducted that in ICU patients undergoing a spontaneous breathing trial, breathing variability is more significant in patients successfully separated from the ventilator and the endotracheal tube [46]. Furthermore, Kiss et al. [47] revealed that VPS was equally effective in weaning as PS mode [47]. However, our findings were discordant with those of Rolland-Debord et al. [48], who claimed that higher breathing variability and lower complexity were associated with higher survival and lower duration of mechanical ventilation [48].

Moreover, there was no apparent difference between the two groups in terms of total days of ICU and overall survival, with p-values of 0.651 and 0.525, respectively. Agreeing with our data, a currently ongoing large multicenter trial examining VPS vs. PS in terms of weaning duration, named the EVA trial (ClinicalTrials.gov Identifier: NCT00786292, [49]) proposed preliminary results that variable compared to conventional PSV didn't increase discomfort or deteriorate the cardiopulmonary function in mechanically ventilated patients in the ICU [49].

In fact, variable PS significantly reduced the work of breathing and increased comfort in some patients. Therefore, the burden and risks to patients resulting from the intervention are low, and patients assigned to variable PS will be weaned faster from the mechanical ventilator than those assigned to conventional PSV. Finally, more trials need to be conducted to further evaluate VPS in terms of more accurate hemodynamic monitoring tools, aeration, patient comfort, desynchrony, and the overall outcome of mechanical ventilation compared to PS.

### Study Limitations

This study has some limitations, including being a single-center study, a relatively limited number of patients, a single tool for hemodynamic monitoring, and being liable for operator bias. Also, multiple factors, apart from mechanical ventilation, could impact hemodynamic status in ICU patients. The parameters of mechanical ventilation were fixed. Therefore, different results could have risen with more diverse parameters. Moreover, no esophageal probe to monitor the mean airway and trans-pulmonary pressures could provide more clues as to whether or not hemodynamic changes are attributed to the different modes. Lastly, many factors can affect weaning success or failure apart from modes of mechanical ventilation.

## Conclusion

Our study found significant hemodynamic effects when transitioning from VCV mode to VPS and standard PS mode, primarily resulting in adverse effects for most patients. Although there was a greater drop in CO among VPS patients compared to those on standard PS, this did not have practical implications for vital signs, particularly mean arterial pressure, or improved clinical outcomes. Additionally, this study concluded that both TTE and TEE typically yield similar results, allowing them to be used interchangeably. Our findings indicate that VPS is as effective as traditional pressure support (PS) in facilitating successful liberation from mechanical ventilation, with no substantial differences in ventilator duration or overall survival rates. The observed hemodynamic stability during VPS, despite some fluctuations, underscores its potential benefits in enhancing patient comfort and reducing the work of breathing. VPS is significantly relevant to the weaning process for mechanically ventilated patients. Lastly, VPS and standard PS led to comparable outcomes regarding ventilator days, liberation success, ICU days, and overall mortality. While more patients were liberated from mechanical ventilation and survived their ICU stay, these differences were not statistically significant. Further studies and trials with large sample sizes are necessary before adopting VPS as a standard method for weaning in conjunction with PSV with an emphasis on more patient-centered endpoints (validated comfort scores).
